# MRI and muscle imaging for idiopathic inflammatory myopathies

**DOI:** 10.1111/bpa.12954

**Published:** 2021-05-27

**Authors:** Samuel Malartre, Damien Bachasson, Guillaume Mercy, Elissone Sarkis, Céline Anquetil, Olivier Benveniste, Yves Allenbach

**Affiliations:** ^1^ Department of Internal Medicine and Clinical Immunlogy Sorbonne Université Pitié‐Salpêtrière University Hospital Paris France; ^2^ Centre de Recherche en Myologie UMRS974 Association Institut de Myologie Institut National de la Santé et de la Recherche Médicale Sorbonne Université Paris France; ^3^ Neuromuscular Physiology Laboratory Neuromuscular Investigation Center Institute of Myology Paris France; ^4^ Department of Medical Imaging AP‐HP Hôpitaux Universitaires La Pitié Salpêtrière‐Charles‐Foix Sorbonne Université Paris France

**Keywords:** idiopathic inflammatory myopathies, MRI, ultra sound imaging

## Abstract

Although idiopathic inflammatory myopathies (IIM) are a heterogeneous group of diseases nearly all patients display muscle inflammation. Originally, muscle biopsy was considered as the gold standard for IIM diagnosis. The development of muscle imaging led to revisiting not only the IIM diagnosis strategy but also the patients’ follow‐up. Different techniques have been tested or are in development for IIM including positron emission tomography, ultrasound imaging, ultrasound shear wave elastography, though magnetic resonance imaging (MRI) remains the most widely used technique in routine. Whereas guidelines on muscle imaging in myositis are lacking here we reviewed the relevance of muscle imaging for both diagnosis and myositis patients’ follow‐up. We propose recommendations about when and how to perform MRI on myositis patients, and we describe new techniques that are under development.

## Graphical abstract

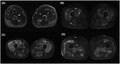

## 1 INTRODUCTION

Idiopathic inflammatory myopathies (IIM) are a heterogeneous group of muscular auto‐immune diseases classified into four categories with distinct outcomes: Dermatomyositis (DM), Inclusion Body Myositis (IBM), Immune‐Mediated Necrotizing Myopathy (IMNM), and Anti‐Synthetase Syndrome (ASyS) (1). The previous group of polymyositis (PM) corresponded to IIM patients without DM skin rash and encompassed IMNM and ASyS or IBM. IIM are characterized by the presence of extra‐muscular manifestations such as skin changes in DM or interstitial lung disease in ASyS, whereas IMNM and IBM patients do not display extra‐muscular clinical signs. While nearly all patients harbor muscle inflammation, the myopathological features vary from one subset of IIM to another, a reason why until recently muscle biopsy was always required for both IIM diagnosis and classification (2).

IIMs treatments combine glucocorticoids and immunosuppressants to induce and maintain disease remission while avoiding or limiting muscle damage. Disease activity, as well as muscle damage, are difficult to assess since clinical evaluation of muscle strength is partly subjective, and reliable biomarkers of disease activity are lacking.

In the past couple of years, ACR/EULAR (American College of Rheumatology and European League Against Rheumatism) both revised IIM diagnostic criteria (3) and proposed a core set of measures to assess disease improvement (4). With the development of myositis‐specific antibodies, they as other scientific societies demonstrated that muscle biopsy is not always necessary for IIM diagnosis (5, 6). However, the use of muscle imaging was not included, either for diagnosis or follow‐up of IIM, as data were lacking to determine its usefulness in IIM.

In this article, we review how muscle imaging provides important information for both IIM diagnosis and follow‐up. We will discuss the most frequently used technique in routine: the muscle MRI, but also other noninvasive techniques (positron emission tomography (PET), conventional ultrasound imaging (B‐mode), functional ultrasound imaging, ultrasound shear wave elastography, and bioelectrical impedance) and their future developments to propose recommendations pending guidelines edited by international workshops.

## 2 MUSCLE MRI FOR IIM DIAGNOSIS

### 2.1 MRI technique in routine

MRI is a non‐invasive and safe technique for muscle exploration. It allows both muscle morphological analysis (e.g., muscle atrophy) and muscle tissue characterization (e.g., fat replacement or edema). The fascia and the skin, also affected in IIM, may also be imaged with MRI.

MRI is a rather long exam. While less than a half‐hour is necessary to assess the lower limbs and the pelvis girdle, a whole‐body MRI (WB MRI) takes around 50 min (7). “WB MRI” without the trunk analysis can be performed in 40 min (7). Of note, MRI of the upper‐limbs can be more time consuming since frequently, depending on patient's size and MRI machine, each limb must be scanned independently. Therefore, most of the studies rely on thigh MRI while a smaller amount of them uses upper limb MRI.

Normal muscle shows intermediate intensity on T1‐weighted sequences and low signal (lower than water or fat) on T2‐weighted sequences (8). For an optimal topographic analysis transverse plane (also known as axial or horizontal plane) is the best orientation.

**FIGURE 1:**
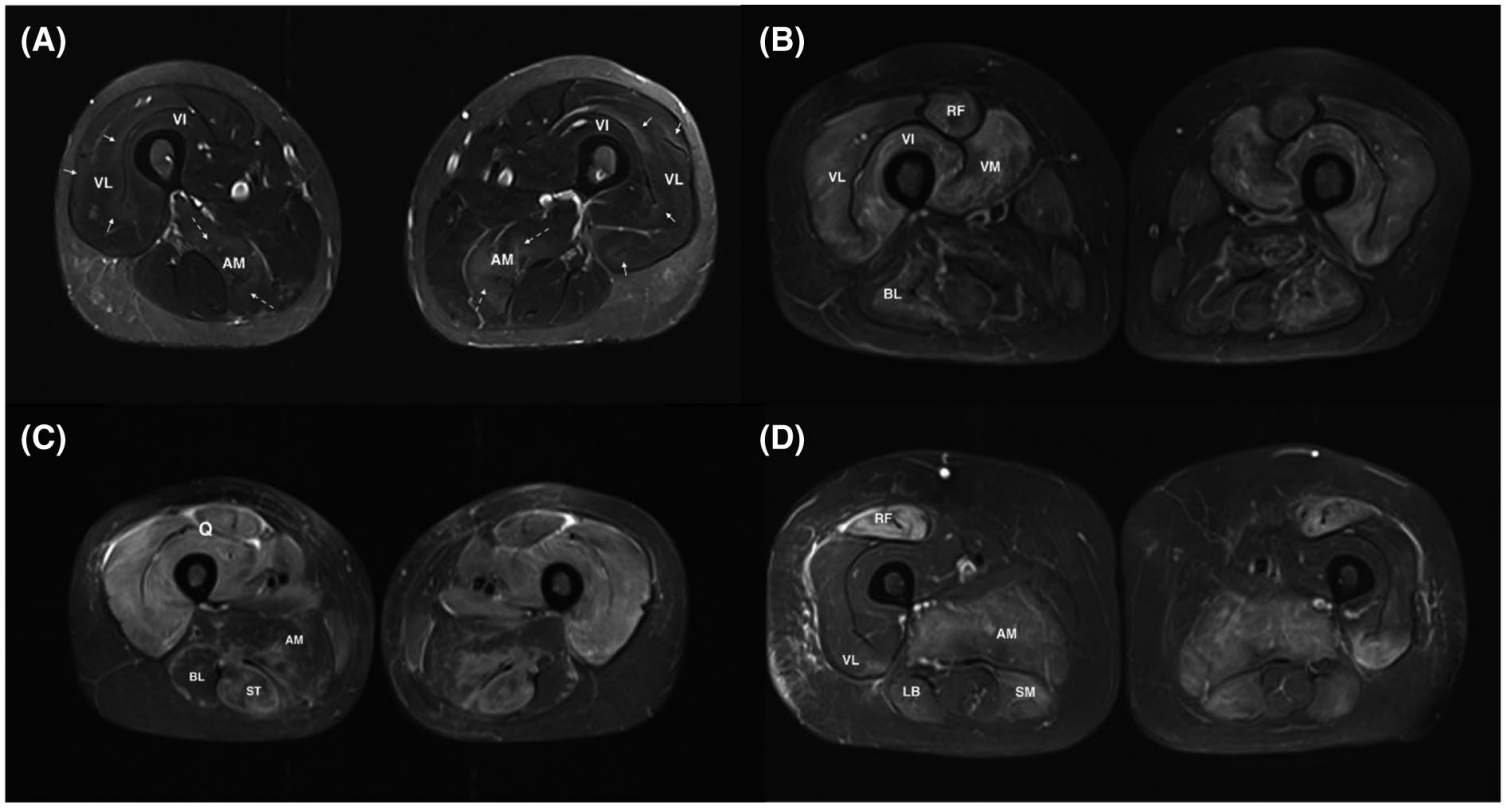
High muscle T2 signals in myositis: (A) Muscle MRI of an overlap myositis (scleroderma) showing mild muscular inflammation. Normal muscles appear with a very low signal (close to the subcutaneous tissue after fat signal suppression). Inflammatory muscles exhibit high signal (slight) with blurred borders, clustered along the aponeuroses and muscular septa (full arrows). The dashed arrows show an area with a more intense hypersignal. (B) Myositis with moderate to severe muscle inflammation. MRI of a dermatomyositis patient showing hyperintense areas affecting mainly the four heads of the quadriceps muscle. (C) ASyS patient with severe muscle inflammation, displaying a marked T2 hyperintensity affecting all three compartments of the thighs on both sides. (D) MRI of an IMNM patient showing that hypersignal is also present when muscle fiber necrosis occurs in absence of significant inflammatory cell infiltratation. All pictures show thigh muscles MRI images (axial plane, T2 STIR w. seq). AM, Adductor magnus; LB, long biceps femoris; Q, quadriceps muscle; RF, rectus femoris; ST, semi‐tendinosus hamstring muscle; VI, vastus intermedius; VL, vastus lateralis; VM, vastus intermedialis

**FIGURE 2:**
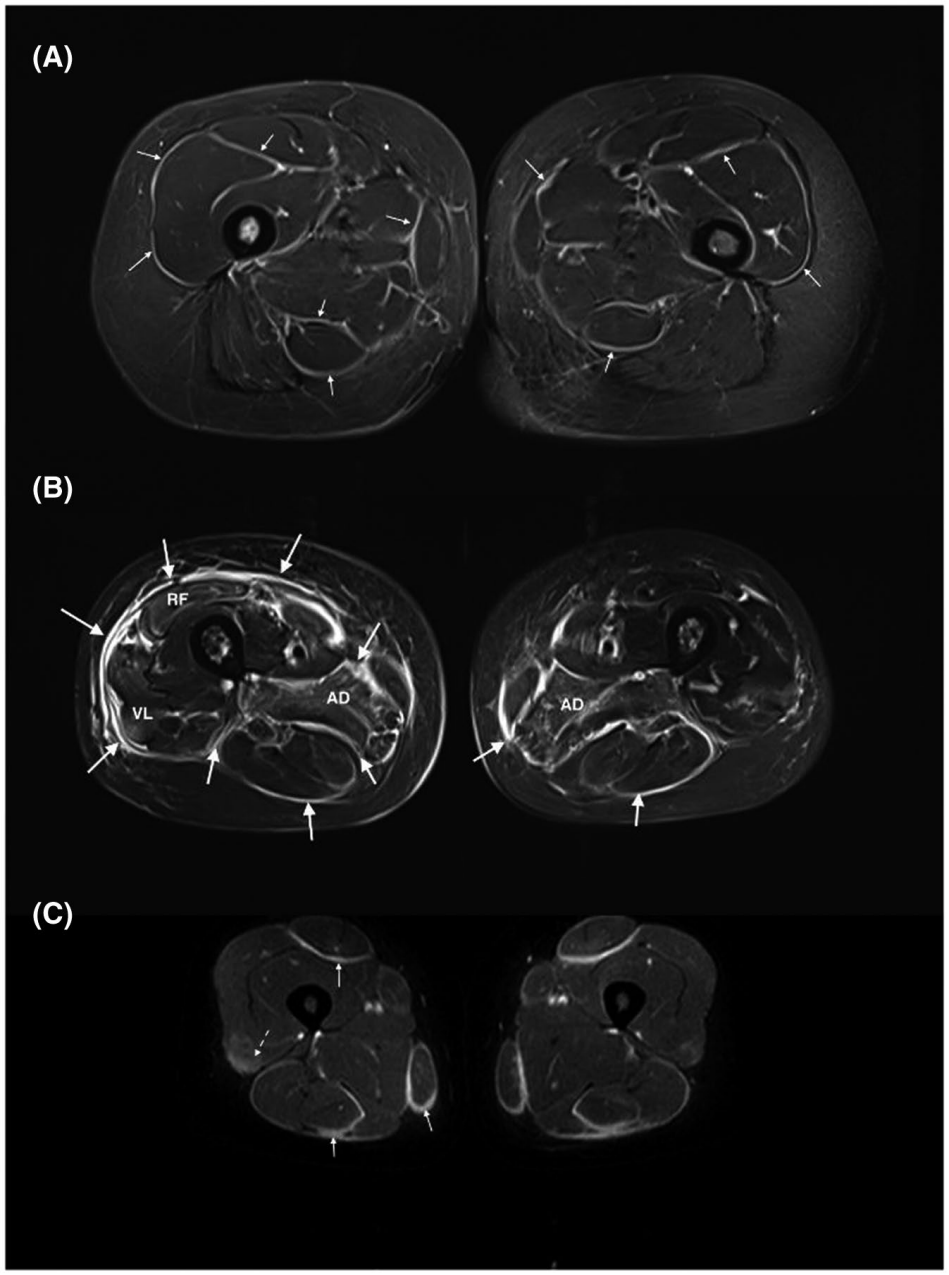
High fascia T2 signals in myositis: (A) patient with an eosinophilic fasciitis displays a diffuse hypersignal of the deep fascia and intermuscular septa (small arrows). The pelvic muscles (i.e., gluteus muscles) are also affected. (B) MRI of a patient with a graft versus host disease involving the fascia (big arrows) (diffuse hyperintensity and thickening of the deep fascia and intermuscular septa) and the muscles (especially both the adductor magnus muscles and the right quadriceps muscle). (C) ASyS patient dysplaying a fasciitis with hyperintense, thickened fascia and intermuscular septa on both sides with symmetrical distribution (full arrows). In addition, presence of a mild myositis attested by a blurred, slight T2 hyperintensity in the quadriceps muscles (dashed arrows). All pictures show thigh muscles MRI images (axial plane, T2 STIR w. seq). RF, rectus femori; VL, vastus lateralis

In routine, specific sequences are needed to detect intramuscular edema, signs of muscle inflammation, or muscle fiber necrosis (9, 10) (Figure 1). T2 sequences show edema as hypersignals more or less homogeneous without mass effect involving muscles and/or fasciae (Figure 2). The best sequences for edema are Short Tau Inversion‐Recuperation (STIR) (11) or DIXON sequences (12, 13). T2 fat suppression sequences are less used in muscular MRIs since the fat saturation is indeed less homogeneous in the usually large fields of views.

T1‐weighted images are used to reveal muscle fatty degeneration and atrophy (14, 15). Atrophy is defined by a loss of muscular volume, but we do not know what is the volume of a normal muscle depending on the age or the sex. Indirect signs can be useful in doubts and when no anteriority is available: thickening of fat tissue located between the muscles, loosening of the muscular aponeuroses and tendons. Fatty degeneration is defined by T1 hypersignal changes of the muscular tissue.

T1 sequence with gadolinium injection also permits the detection of edema. This sequence does not have a better sensitivity than the STIR sequences (16, 17) for inflammation detection (Figure 2). Permanent gadolinium deposits in the brain may occur after injection (18, 19) leading to hypersignals of the dentate nucleus and globus pallidus years after injection (20). The long‐term consequences remain unknown. For muscle MRI in the case of IIM, sequences with gadolinium injection are not recommended. However, they can be useful to detect a fasciitis or to help characterize a focal myositis (21, 22).

### 2.2 MRI and muscle inflammation at IIM diagnosis

Muscle biopsy is an invasive technique for IIM diagnosis but it is the gold standard. In addition, it allows subgrouping myositis patients into four basic categories (1) and even beyond (5, 23).

Recently, ACR/EULAR revised IIM diagnostic criteria and demonstrated that it can be possible to diagnose an IIM without myopathological analysis using a combination of clinical and biological data (3). In addition to clinical items (e.g., muscle strength or skin manifestations), ACR/EULAR proposed to use muscle enzymes and myositis‐specific antibodies (i.e., that are present in ~70% of IIM) as non‐invasive biological tests (24). Creatine kinase levels are not specific and may be normal in up to 30% of cases especially when muscle fiber necrosis is absent such as in DM (25, 26). Myositis‐specific antibodies are specific but only anti‐Jo‐1 antibodies were included in ACR/EULAR criteria and are present in a minority of IIM patients.

MRI has not been included as a diagnostic technique even though the sensitivity for IIM diagnosis was good. One explanation is that few studies reported the sensitivity of MRI for IIM diagnosis: previous studies reported 90% sensitivity (17, 25) for IIM diagnosis. Thigh muscle MRI showed 86% sensitivity for DM/PM (27), 66% for ASyS (28), 83% for IMNM (15), and 72% for IBM (29).

MRI diagnostic performance could depend on the analyzed region. Thigh muscle MRI may be more relevant for IMNM since muscles of the lower limbs are predominantly involved in this condition (15, 30, 31). Though, muscle strength deficit is frequently more severe in the upper limbs in DM (1). Most IIM patients (99%) with hyperintense signal (inflammatory muscular edema) display thigh muscle involvement that is the most frequently affected area on whole‐body MRI (WB MRI) in DM or PM (27).

On the other hand, WB MRI tends to show a better sensitivity for IBM (32, 33). To the best of our knowledge, no data are describing WB MRI diagnostic performance for ASyS or IMNM.

Furthermore, MRI can also detect subclinical muscle inflammation such as in amyopathic DM (34), in which up to 100% of patients have muscle inflammation on WB MRI, or in amyopathic ASyS patients where muscular inflammation is frequently observed (28).

### 2.3 Muscle MRI and muscle biopsy for IIM diagnosis

MRI sensitivity is good but not perfect: pathological analysis can exhibit inflammatory infiltrates in muscle areas without any hyperintense signal on MRI (35) demonstrating that the muscle biopsy remains the most sensitive technique. Besides, muscle biopsy is crucial for IIM classification as only myopathological findings are specific to the IIM subsets (5, 23).

On the opposite, when a muscle biopsy is required for IIM diagnosis, it may show false‐negative results in up to 10%–20% of cases (26, 27), probably because of sampling errors, non‐specific changes, or the predominance of fat tissue within samples (muscle degeneration).

To improve the sensitivity of the biopsy some authors have suggested that the biopsy could be guided by MRI (36, 37, 38, 39). One study compared clinical‐guided versus MRI‐guided muscle biopsies suggesting the significance of pre‐biopsy MRI, but the low reported sensitivity of clinical‐guided biopsy is unusual (38) as compared to other studies (80%) (26, 27). Anyway, MRI guidance has limitations. First, delimiting, during the MRI, a target muscle, and applying a mark on the area containing the muscles to biopsy may be difficult and/or time‐consuming. Second, the targeted muscle area may not always be accessible for a biopsy if it is a deep region especially when an open biopsy is planned (40). It must also be reminded that the biopsy may show inflammatory infiltrate even if the muscles do not show a hyperintense signal (35).

It turns out that the choice of a muscle to biopsy is easier and more efficient based on the clinical examination and the EMG findings (NOTE: the muscle biopsy has to be taken from the contralateral side after EMG exam to avoid needling artifacts! This procedure should be feasible for most of the symmetric muscle diseases) for the large majority of patients (41).

### 2.4 Muscle MRI and IIM classification

The pathological (microscopic) muscle image analyses allow IIM diagnosis and classification (3) whereas this is not possible with muscle MRI. Nevertheless, a muscle biopsy allows the analysis of a few milligrams piece of skeletal muscle whereas WB MRI allows the analysis of dozens of kilograms of muscle issue that represents approximately 40% of total body weight (42). In addition to muscle, MRI allows exploring of fascia, skin.

Regarding the muscle compartment, the topography of the muscle lesions depends on the IIM subsets.

#### 2.4.1 IBM

This is especially true for IBM where clinical flexor digitorum profundus and quadriceps involvement is characteristic for the disease and is included in the diagnostic criteria (43). At the beginning of the disease, the finger flexors and/or quadriceps involvement may not be clinically detectable, whereas it may already be visible on MRI.

Muscle inflammation is frequent in IBM, up to 78% on WB MRI, and is present in most muscles but is usually sparse (32). This inflammation tends to be asymmetric. Compared to other IIM it is more frequently in the anterior muscles of the thigh and forearm, and the distal part of thigh muscles (29, 32, 33).

Beside muscle inflammation, IBM patients display signs of muscle degeneration at a microscopic level (e.g., rimmed vacuoles, protein aggregates, atrophic fibers, and fat cell replacement in muscle biopsy) and at a macroscopic level as the large majority of IBM patients also show muscle atrophy and/or fat replacement on MRI.

In lower limbs, muscle fatty degeneration is predominant in the anterior and distal part, with relative sparing of the rectus femoris muscle (32, 33, 44). In the legs, changes are prominent in the gastrocnemius muscles with relative sparing of the tibialis posterior and the soleus (17, 32, 44). In the upper limbs, the most affected muscle is the flexor digitorum profundus (32, 44). Pelvic muscles are always less involved than thigh muscles (33). Finally, a study demonstrated that a typical IBM pattern defined as fatty‐fibrous infiltration and atrophy of both quadriceps muscles in the distal portion (vastus intermedius and medialis muscles) has a sensitivity of 80% and specificity of 100% for IBM diagnosis (33).

#### 2.4.2 IMNM

As IBM, IMNM can be considered as a muscle‐specific autoimmune disease. IMNM can also present with a characteristic muscle phenotype that is less specific than that in IBM. Clinically, it was shown that IMNM patients have more severe muscle weakness predominantly involving the lower limbs (1, 45). Accordingly, thigh muscle MRI shows more extensive edema in IMNM compared to that in DM or PM (15, 46). Besides, it was demonstrated that IMNM also has more fatty replacement and atrophy especially on the lateral rotator and in the glutei muscles (46, 47, 48).

#### 2.4.3 DM

Muscle MRI studies in DM also suggest a characteristic MRI pattern. Clinically, muscles of the upper limbs are more severely affected than those in the lower limbs (1). Muscle MRI reveals symmetrical involvement predominant in the pelvic and shoulder girdles (46, 49). Furthermore, one study showed that a high signal intensity in STIR images (as well in gadolinium‐enhanced fat‐suppressed T1‐weighted images) organized as a heterogeneous reticular “honeycomb pattern” was characteristic of DM patients (50). The high signal intensity is also suggestive of DM when showing a peripheral distribution in the muscles (27, 46, 50). DM patients exhibit more frequently high signal intensity in fasciae compared to that in other IIM (46, 49, 50, 51), but also in subcutaneous tissue (46, 49, 50, 51).

Combining subcutaneous, fascial high signal intensity, peripheral distribution, and honeycomb pattern, Ukichi et al. (50) developed a score for DM diagnosis showing good sensitivity and specificity. Nevertheless, since the majority of DM patients exhibit characteristic skin rashes and antibodies, the usefulness of muscle MRI to classify IIM patients in DM would be only relevant for DM sine dermatitis and without DM‐specific antibodies (a very rare condition) (52).

#### 2.4.4 ASyS

The muscle MRI pattern of ASyS patients is less known since most studies did not isolate this subset from DM or PM. Two independent studies have shown that in addition to thigh muscle edema, patients frequently have high signal intensity in fasciae (28, 50) (Figure 2).

### 2.5 MRI for IIM prognosis and extra‐skeletal muscular complications

Life‐threatening complications in IIM are mainly linked with extra‐muscular complications (53). Malignancy, lung involvement, and cardiovascular diseases are the main causes of mortality in IIM.(53) The presence and the type of myositis‐specific antibodies are crucial for identifying patients likely to develop a rapidly progressive interstitial lung disease (e.g., anti‐MDA5) or a malignancy (e.g., Anti‐TIF1‐γ), but do not permit to predict with certainty such complications.

It was shown that among DM, including amyopathic patients, a predominant fascia involvement was an independent risk factor for rapidly progressive interstitial lung disease (54).

Some authors (27) argued that WB MRI could assess the whole body musculature and detect neoplasia at the same time. Unfortunately, oncologic and muscular WB MRI requires a very different protocol, as T1, STIR or DIXON T2, and diffusion sequences are mandatory in oncology. Besides, muscular WB MRI is usually acquired on an axial plane, unlike oncologic WB MRI.

“WB MRI” can be done without imaging the trunk in IIM and shows no statistical difference of detecting inflammation when compared to “true WB MRI” (7).

WB MRI is not an all‐in‐one exam for IIM, since specific protocols are needed depending on the objectives.

The same holds true for myocarditis associated with IIM. Cardiac MRI need specific protocols different from those developed for skeletal muscle.

### 2.6 Limitations of MRI

#### 2.6.1 High T2 muscle signal definition

In routine, MRI image analysis is qualitative and definition of hyperintense signal is mostly subjective. Hypersignal is defined relatively to a seemingly normal tissue (Figure 1), partly explaining why the reliability is not perfect. Nevertheless, studies showed a good inter‐observer agreement for muscle edema detection using STIR or gadolinium‐enhanced fat‐suppressed T1‐weighted imaging with variations depending on the muscle groups (50, 55). The inter‐observer agreement was moderate for axial muscle and almost perfect for some proximal muscle such as gluteus muscle (55).

#### 2.6.2 High T2 muscle signals specificity

**FIGURE 3:**
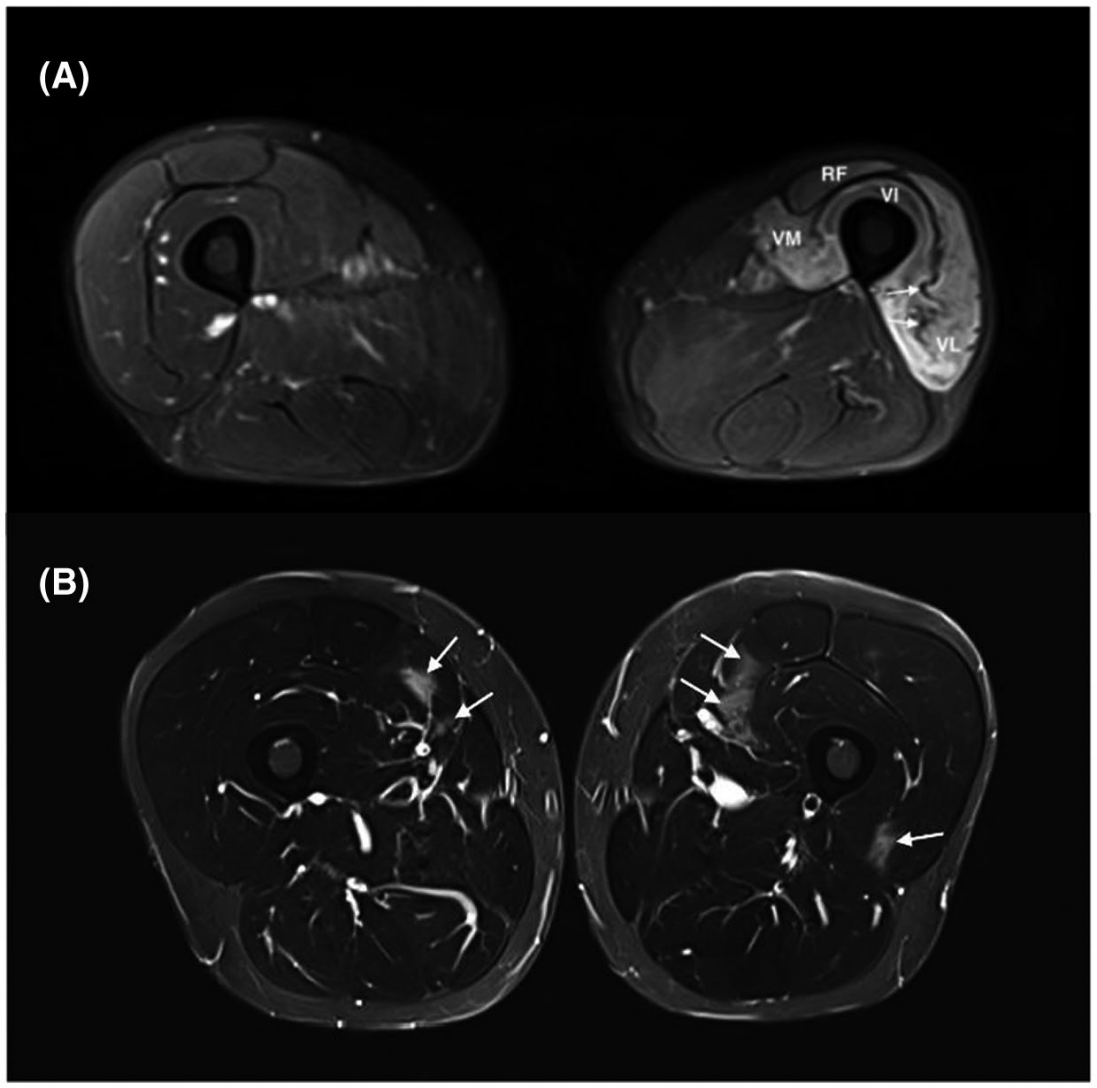
High muscle T2 signals in non‐myositis patients: (A) Neurogenic muscle edema was observed on an MRI performed 4 months after an iatrogenic lesion of the femoral nerve (inguinal hernia surgery). Intense signal and atrophy of the left quadriceps corresponding to the femoral nerve territory are present. Atrophy can be easily identified as compared to the contralateral thigh. Slackness of intramuscular septa (arrow) can also give a clue. (B) MRI 24 h after intense exercise showing patchy areas with increased T2 intensity affecting the quadriceps muscle (arrows), predominantly its medial head, with normal muscle biopsy and spontaneous remission of muscle signs and CK elevation. All pictures show thigh muscles MRI images (axial plane, T2 STIR w. seq). RF, rectus femoris; VI, vastus intermedius; VL, vastus lateralis; VM, vastus medialis

Myopathological changes are specific for IIM diagnosis whereas muscle MRI abnormalities are not specific (Figure 3).

Several conditions may induce high signal intensity on T2 weighted images in the absence of muscle inflammation. Simple artifacts can induce false‐positive results. For instance, there is a frequent moderate hypersignal on the distal regions of medial gastrocnemius at their attachment over the soleus muscle. Chemical shift artifacts can induce false hypersignals next to the fasciae (56). In damaged muscles and according to the fat suppression technique used on the T2 images, muscular tissue degenerated by fat can show a slightly more intense signal than the normal muscular tissue, making the distinction from mild muscular edema difficult.

High signal intensity may also be induced by muscle exercise (57) (Figure 3). In physiological settings, it is established that normal muscles show an increased signal intensity on T2‐weighted images during and after exercise. This high signal intensity returns to baseline between 10 min and 1 h after the end of exercise (58, 59, 60, 61, 62).

Along that line, Summers et al. asked 32 patients with juvenile DM to climb repeatedly a single step for a mean time of 6 min before MRI and observed hypersignals in STIR sequences for the majority of the patients (63). Exercise‐induced STIR signal may occur in muscles with normal baseline signal and return to baseline values 30 min after the exercise (63). Of note, the muscular distribution of the exercise‐induced signal was similar to those induced by muscle inflammation showing that exercise may cause MRI false positivity in IIM (63).

In pathological conditions, MRI may also show a high signal in muscles in case of vascular diseases such as deep vein thrombosis (64), sickle cell crisis (65), and muscle infarction in diabetic patients (66, 67). Intrinsic and extrinsic traumata are frequently associated with muscle edema (68). In particular, intense muscle effort may induce a Delayed Onset Muscular Soreness (DOMS), with muscular edema and hypertrophy on MRI (Figure 3). Iatrogenic myositis with edema can happen in the field of an external or internal radiation therapy. Infectious myositis or pyomyositis is revealed by a diffuse inflammation and often a collection of pus (69). A pseudo‐tumoral edema can also be induced by ossifying myositis (70) or focal myositis (21). In these conditions, the key point is that muscle MRI changes are unilateral and usually localized in a muscle or a group of muscles.

Other inflammatory diseases may have a muscular presentation. Of these, sarcoidosis can show three different patterns: nodular with pseudotumors on MRI (71) which is the most typical one, acute with pain and increased CK (72), or chronic (73). Moreover, systemic lupus erythematosus (74), systemic sclerosis or overlap syndrome can give muscular inflammation, but their presentation is similar to IIM (75). Vasculitis can also involve muscles.

Non‐inflammatory muscular diseases like Statin‐induced myopathy and muscular dystrophies can show some extent of muscular MRI T2 hypersignal, even though muscular atrophy and fatty degeneration usually predominate.

Finally, muscular hypersignal on T2 and STIR sequence may be also observed after only 4 days of denervation (76, 77, 78), in any nerve injury such as Parsonage–Turner syndrome, amyotrophic lateral sclerosis, acute motor axonal neuropathy, or lumboradiculopathy (Figure 3). The key point is that the denervation edema usually follows a radicular or a truncal distribution. These observations highlight the interest of electromyography to differentiate myopathic from neurological injuries in case of muscle hyper‐signals of an unknown origin. Lastly, swelling and edema in paravertebral muscles can be observed before fatty involution in Parkinson's disease with camptocormia (79).

High T2 muscle signals are not specific for inflammatory infiltrates.

## 3 MUSCLE MRI FOR IIM FOLLOW‐UP AND MUSCLE DAMAGES ASSESSMENT

IIM disease activity may be difficult to assess especially in patients with longstanding diseases. The distinction between sustained muscle activity and muscle damage is crucial for decision‐making, including therapeutic strategy.

To analyze treatment efficacy, the ACR/EULAR developed an improvement score combining core set measures including parameters for muscle domain assessment such as muscle enzymes or muscle strength evaluation (manual muscle test 8 score) (80, 81, 82). The total improvement score is designed to capture only an improvement between two time points and not to determine disease activity at one time point.

In addition, one must recall that CK can be normal in up to 30% of DM (26) and that the reliability of manual muscle testing (MMT) has some limitations (83). Other methods can be used for disease activity assessment of myositis such as EMG which showed a good sensitivity and specificity (27, 84), or muscle biopsy which is, however, invasive.

### 3.1 Muscle MRI and disease activity assessment

Muscle MRI is an important technique for evaluating disease activity. MRI studies demonstrated a correlation between the amount of inflammatory infiltrates within muscle biopsies and the intensity of STIR hypersignal (25, 35, 51).

In patients with an active disease, especially in patients with high CK levels, STIR hypersignals (semi‐quantitative assessment) are significantly higher than in patients with inactive disease (9, 28, 55, 85, 86). Most IIM patients with inactive disease have normalized their MRI signals (9, 55, 85).

In DM, muscle STIR signal correlates with muscle strength and the different clinical scores of disease activity (55). However, some patients with inactive disease (normal CK level and manual muscle testing) may have persistent hypersignals (27, 28, 85) suggesting a subclinical disease activity.

Usually, 6 to 10 weeks are necessary for CK normalization (87) and muscle biopsies do not show any sign of inflammation 6 months after treatment onset (87). Concerning muscle MRI, only few data are available. Previous studies reported persistent MRI hypersignals 1 or 2 months after treatment initiation (88, 89, 90), then decreasing significantly after 3 months (91).

In DM, the sensitivity of thigh muscle MRI for muscle disease activity was estimated to be 90% (85). In IMNM, STIR hypersignals were associated with a good response to treatment (15, 31), along with CK levels, which proved to be a very good marker for disease activity (92).

Muscle edema (STIR) in IBM is frequent (78%) (32), but it is not associated with disease duration, CK level, or muscle strength (29, 32, 46). Other parameters, such as muscle atrophy or fatty replacement, may be involved in IBM muscle weakness.

Together those data suggest that muscle disease activity correlates with high T2 signal, but in routine circumstances, high signal definition is subjective as well as is its rating (semi‐quantitative assessment).

### 3.2 Muscle MRI and muscle damages

**FIGURE 4:**
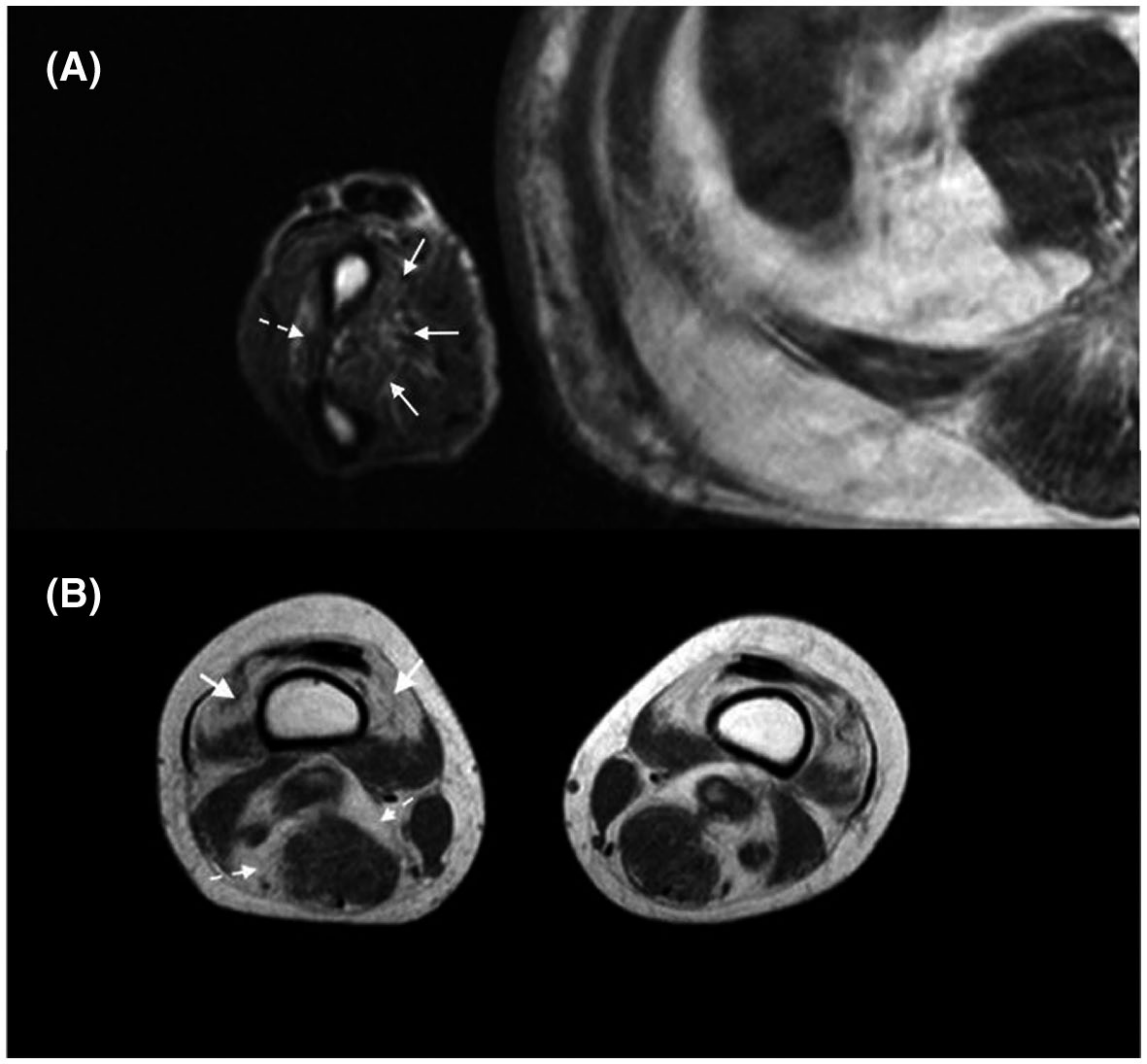
MRI muscle damages in myositis patients. (A) Upper limbs muscle damages in inclusion body myositis attested by a mild fatty replacement of the muscles of the forearm, involving predominantly the deep flexor digitorum (full arrows), and to a lesser extent the extensors (dashed arrows). (B) Lower limbs muscle damages in inclusion body myositis attested by a distal involvement encompassing muscle atrophy (loss of volume with the widening of the fat tissue between muscles, dashed arrows) and fatty replacement (muscular T1 hypersignal) occurring mainly in the quadriceps femori muscle (full arrows). All pictures show axial plane, T1 w. seq of the arm A or the thigh B

Persistent muscle inflammation may lead to permanent pathologic changes including muscle atrophy and/or fatty replacement (Figure 4). These muscle damages can be detected on T1 weighted images without fat saturation (30, 90) in order to assess muscle fatty degeneration and atrophy (14, 15).

In muscle damage, atrophy is more difficult to diagnose and assess in the absence of normative muscle size for a given muscle at a given age and sex, especially if the disease has a bilateral or diffuse pattern. Nevertheless, atrophy is usually absent when DM is diagnosed (27), but occurs in 14% of cases during follow‐up (27). In IMNM, it is reported in 23% of cases and it is interestingly associated with STIR hypersignal (46). Almost all patients with IBM display muscle atrophy at diagnosis, but less diffusely than fat replacement (29, 32) (Figure 4).

Regarding fatty degeneration, in routine conditions, only semi‐quantitative methods have been proposed to minimize interobserver variation, among which the most commonly used are Goutallier (93) or Mercuri scores (30). Fatty infiltration increases with IIM disease duration (14, 15, 27, 30, 46). In none of IIM, it is associated with CK elevation (14, 27, 46).

At diagnosed IIM, 14% of patients diagnosed as having PM and DM patients display muscle damage on MRI (27), and almost 100% of IBM or IMNM patients have fatty infiltration and atrophy (15, 29, 32). In the course of IIM follow‐up, fatty infiltration occurs in half of “PM” and DM (27, 85) and in 42% in ASyS (28).

In IMNM and IBM, atrophy and fatty replacement develop at the “early stages” of the disease (15, 27, 46).

Along that line, it was demonstrated that IMNM is more severe in terms of muscle weakness as compared to that in other IIM (46, 94). Besides, IBM is slowly progressive, around 3% per year increase in muscle fatty replacement (14), to the extent that the diagnosis (and muscle MRI) is usually delayed by almost 5 years after the onset of the first symptoms (95).

IBM and IMNM have the highest load of lesions (46), it is similar in both diseases after ten years of evolution (30), but its distribution is different. IMNM tends to show mostly involution and atrophy in the pelvi‐femoral muscle group, shoulder region, and lumbar region (15, 30, 47), whereas in IBM, the damage is predominant in the anterior and distal part of the thigh, and anterior part of the arm. Quantitative MRI allows to measure precisely damage progression, and most importantly, muscle fatty replacement is correlated to muscle strength but also to functional scales in IBM (14).

Regarding IMNM, the increase in fatty replacement is probably associated with a poorly controlled disease and a poor response to treatment (15). In addition, the percentages of fat replacement correlate with the muscle strength (14, 30, 32) showing that it is a good surrogate marker of muscle damage.

Finally, as mentioned above, WB MRI can be helpful for IIM classification based on hypersignal distribution, but for IIM follow‐up, MRI of the thighs is the most sensitive procedure to detect the evolution of the disease (96) and is probably sufficient to assess the evolution of disease damages. Indeed, most studies showed a predominant thigh muscle involvement, in both DM and PM (27) and 100% in IMNM and IBM (30).

MRI is probably one of the best techniques to assess muscle damage including muscle atrophy and fatty replacement. Without precise muscle atrophy definition, only significant muscle atrophy can be reported. Fatty replacement can be measured very precisely, but in routine situations, only semi‐quantitative analysis is used (lack of sensitivity).

### 3.3 Limitation in muscular damage assessment

As hypersignal STIR is not specific for muscle edema in IIM, fatty replacement and muscle atrophy are not specific for IIM muscle damages.

During the course of IIM, even if muscle damage is mainly caused by IIM, muscular atrophy may not be linked to the disease itself but to the therapeutic complication such as steroid myopathy which frequently involves the quadriceps (97, 98).

Degenerative damage frequently occurs during aging (99, 100). Thus, muscle MRI of healthy controls with a mean age of 62 years showed around 5% of fatty infiltration in thigh and calf muscles (14).

Prolonged immobilization and rupture of tendons usually induce atrophy and fatty degeneration.

Furthermore, all the diseases affecting the muscles and listed in the previous paragraph (traumatic, vascular, infectious, inflammatory, etc.) can induce muscle damage, especially if they are chronic. Similarly, Duchenne muscular dystrophy (101), myotonic dystrophy (102), or desminopathy are associated with fatty replacement. After muscular edema (78), irreversible muscular denervation induced fatty replacement, for example, in Charcot Marie Tooth disease (14). Parkinson's disease (79) is also associated with fatty replacement.

## 4 MUSCLE IMAGING DEVELOPMENT

In addition to MRI routinely performed, other muscle imaging techniques are in development besides the improvement of MRI sequences.

### 4.1 MRI

#### 4.1.1 Quantitative MRI

To bypass MRI limitations, quantitative analysis is indeed crucial. Advances in imaging techniques have made the quantification of T1 and T2 and to generate color‐encoded T1/T2 maps, in which the pixel values represent the T1 and T2 relaxation time actual value in each voxel feasible (rather than a signal relative intensity in arbitrary units).

In juvenile DM, a good correlation between MRI T2 relaxation score and disease activity was found (9). In IBM, clinical weakness is correlated with data from quantitative MRI (14, 103, 104). Results from quantitative MRI were comparable to the semi‐quantitative assessment of edema by a trained radiologist (12). For fatty infiltration, quantitative MRI seems to be more precise and reliable (105).

The method for relaxation time measure is not yet standardized, and normal values from a large healthy cohort are missing (103), but clearly quantitative MRI appears as the most promising technique.

#### 4.1.2 Diffusion sequences

Diffusion‐weighted Imaging (DWI) allows to analyze random water movement in tissue in order to help determine tissue structure. This can be quantified by the apparent diffusion coefficient (ADC). Several studies have analyzed ADC in muscle from myositis patients (11, 106, 107) showing higher values in edematous muscles, but also some muscles which did not show T2 hypersignals may suggest a better detection of low‐grade edema (11). Other studies will have to be undertaken to determine the input of these specific MRI sequence techniques in IIM. Diffusion could also be useful for monitoring the disease as ADC is augmented in active myositis and reduced in case of fatty infiltration (106).

Tractography can be extracted from specific, multidirectional diffusion sequences, mainly on 3 T MRI, allowing colored maps and 3D mapping of anisotropic structures like nerves. Some attempts have also been made on muscles with various success (108).

#### 4.1.3 MRI 3 Tesla vs 1.5 Tesla

In these past few years, high field MRI at 3.0 T has shown its superiority over 1.5 T in neurological imaging. For muscle MRI, this superiority has not yet been demonstrated. 3.0 T imaging has a better signal over noise ratio, but induces more artifacts (109, 110, 111).

#### 4.1.4 Phosphorus 31 magnetic resonance spectroscopy

Magnetic resonance spectroscopy using phosphorus 31 is a research technique developed in the 70 s to explore muscle metabolism. It is based on the evaluation of the ratio between inorganic phosphorus and phosphocreatine or ATP (112). In IIM, studies with this technique are rare. They reveal an increased ATP consumption during efforts but no relation between ATP consumption and inflammation intensity (13, 90, 112).

### 4.2 Technics of nuclear radiology

#### 4.2.1 [18F] fluorodeoxyglucose positron emission tomography/computed tomography (FDG PET)

The increased risk of cancer is well established in IIM (113, 114, 115, 116, 117) and FDG PET is frequently used for cancer screening (118, 119).

FDG PET imaging also permits to detect muscle inflammation, but normal values for muscle Standardized Uptake Value (SUV) are difficult to define (120, 121, 122, 123). The sensitivity for muscle inflammation is broad: 33–90% (120, 121, 122, 124) in IIM depending on muscle inflammation definition (qualitative or quantitative definition). Muscle SUV seems correlated to CK levels and MMT (124, 125), and one study found a correlation between inflammation on biopsies and SUV (124).

When compared to a validated technique for assessment of IIM activity, FDG‐PET showed that muscle FDG uptake measurement has high sensitivity and specificity to distinguish active from non‐active muscle disease and a good sensitivity to detect changes in muscle disease activity (122).

Studies comparing MRI and FDG PET sensitivities for muscle edema are too rare to draw conclusions (121, 124).

Of note, [18F] Florbetapir, an agent used for amyloid enhancing on PET, was tested to differentiate IBM from PM and showed a good sensitivity of 80% and a high specificity of 100% for IBM in a pilot study (126).

### 4.3 Ultrasound imaging in IIM

Amongst soft tissues, skeletal muscles are particularly suitable for being examined with ultrasound imaging (US). US has the advantages of being accessible, cheap, and portable. One of the main limitations of US is its strong operator‐dependency as compared to MRI.

#### 4.3.1 Conventional B‐mode US

US can detect typical myositis features such as edema, calcifications, atrophy, fascial thickening, and degenerative processes (i.e., fibrosis, fatty infiltration) (127, 128, 129). Inflammatory muscle fascicles appear as hyperechoic surrounded by fibroadipose septa filled by inflammatory exudates with hypoechoic appearance (130). MRI is probably better suited to detect edema (131).

In IIM, there is a slight decrease in echogenicity in the acute phase while in the chronic phase, echogenicity increases and is accompanied by a reduction in muscle cross‐sectional area/ thickness. In DM, an increase in echogenicity can be focal with a “see‐through appearance,” associated with an increased echogenicity of subcutaneous tissue (132). In IBM, echogenicity is substantially increased with lowered muscle cross‐sectional area/thickness. Involvement may be asymmetric. The muscle may also appear as “moth‐eaten” (133).

Echogenicity is most commonly assessed using a visual scale proposed by Heckmatt et al. (134), but to bypass the limitation of semi‐quantitative scale limitations, computer‐assisted quantification of mean echogenicity is extensively used (135). Though quantification of echogenicity offers obvious advantages, echogenicity also shows gender differences and a muscle‐specific non‐linear relationship with age (136), and there are no standardized reference values (137). Beyond mean echogenicity, other first‐order descriptors (e.g., standard deviation, skewness, kurtosis, and entropy) and higher‐order texture features have been identified as interesting parameters for characterizing and monitoring muscle degenerative changes (138, 139, 140, 141, 142, 143).

Another challenge is that echogenicity may be decreased at acute stages with edema and increased at chronic stages with fibrosis and fatty infiltration. Assessment of IIM specific patterns of muscle involvement may be performed (144, 145). Other approaches relying on “deep learning” may contribute to improving the specificity and sensitivity of muscle US in IIM for both diagnosis and follow‐up (146, 147).

Further studies comparing US and MRI findings, in particular during longitudinal studies of US, are required to better understand the potential of US in IIM.

#### 4.3.2 Functional US

Standard power Doppler US for assessing changes in muscle vascularity/perfusion is limited in IIM as muscle Doppler signal is absent or very low at rest (148). Contrast‐enhanced US is more sensitive to blood flow within capillaries, allowing the assessment of muscle perfusion (149).

In PM and DM, an increase in muscle perfusion was reported in patients with edema on MRI and histologically confirmed diseases (150). Contrast‐enhanced US may be helpful to guide diagnosis and biopsy planning as increased perfusion associated with non‐specific muscle edema is typical in active myositis (151). Data clarifying the usefulness of contrast‐enhanced US in IIM remain sparse.

#### 4.3.3 US elastography

Ultrasound elastography techniques provide an opportunity for direct quantification of tissue elasticity or stiffness (152) that may be affected by structural alterations induced by disuse and pathological processes (153). Elastography may be used to assess muscle at rest, during contraction (154), or passive stretching (155). The most recently developed method, namely US shear wave elastography has been extensively used in the skeletal muscle. IIM may combine atrophy, fatty infiltration, fibrosis, and edema. It is unclear whether these changes affect passive mechanical muscle properties when they co‐occur (156). Many factors influence muscle stiffness (e.g., muscle length, muscle three‐dimensional structure, tendon compliance, muscle activation, muscle perfusion, edema, fat content) (153).

In IIM, using compression elastography, early studies reported increased muscle stiffness at rest in patients with active myositis (157). In juvenile IIM, active myositis was not accurately detected with compression elastography (158). In IBM using shear wave elastography, lower muscle stiffness has been shown to be associated with more severe muscle weakness (159). A recent study also showed lowered muscle stiffness in patients with IIM that was associated with muscle weakness and MRI scores of edema (160). Given these discrepancies, further research is needed. A critical point is that studies that have fundamentally investigated relationships between local muscle elasticity and the severity of degenerative muscle damage (which is the most relevant potential use of shear wave elastography in IIM) as assessed with MRI and/or biopsy analysis are particularly scarce (155). Other approaches such as shear wave spectroscopy for the assessment of muscle viscosity (161, 162) or sound speed estimation for estimating fat content (163) may potentially provide useful biomarkers for IIM in the future.

### 4.4 Bioelectrical impedance methods in IIM

Bioelectrical impedance refers to all methods based on the characterization of the passive electrical properties of biological tissues in response to the application of an external current (164).

A limitation of traditional bioelectrical impedance analysis approaches is that they are using descriptive models relying on poorly generalizable sample‐specific regression equations (164, 165, 166). However, traditional bioelectrical impedance analysis may be useful for assessing and monitoring overall muscle mass and adiposity in IIM. Recently in IBM, a bioelectrical impedance analysis method based on serial bioelectrical measurements was shown to provide accurate estimates of lean thigh muscle volume versus quantitative MRI (167). This method may be useful to monitor the effects of treatment on lean muscle volume although further studies are needed to investigate its sensitivity to changes in muscle disease activity. Other approaches, termed electrical impedance myography and needle impedance myography, aim at locally investigating the specific bioelectrical properties of muscle and are currently investigated (168, 169, 170, 171).

## 5 CONCLUSION

Muscle imaging and especially muscle MRI is a powerful technique for both IIM diagnosis and follow‐up. Muscle MRI is actually the best muscle imaging technique in routine, and it is widely used, but guidelines determining when and how to use it in myositis patients are lacking.

Thigh muscle MRI is a good technique for IIM diagnosis, but it must be used in combination with clinical and biological (i.e., CK and muscle specific‐antibodies) parameters to limit its lack of sensitivity and specificity and to reduce the use of muscle biopsy. Thigh muscle MRI is also useful to follow patients to differentiate muscle disease activity from muscle damages. Muscle MRI is a good biomarker of both disease muscle activity and muscle damage since hyper‐intense T2 and fatty replacement have been correlated to muscle strength. It appears that the main limitation of muscle MRI is the definition and the assessment of MRI changes. To overcome this difficulty MRI quantitative measures or the development of new techniques are necessary.

**Muscle MRI recommendations in IIM proposed by the authors**

Why performing an MRI?

1. MRI may be useful for IIM diagnosis but also to rule out IIM diagnosis (absence of hyperT2 signal) in combination with clinical signs, CK level, and myositis‐specific antibodies
2. Only in specific cases, MRI is useful to guide the muscle biopsy when the muscle biopsy is required (e.g., myopathic patients with important muscle damages)
3. In case of IBM suspicion, MRI may exhibit characteristic changes, in other cases, MRI is not recommended to classify IIM patients into myositis subgroups
4. MRI is important in IIM follow‐up to help to discriminate muscle damages from muscle disease activity when other muscle activity disease biomarkers are missing

How to perform an MRI?

- Pelvic and thigh muscles analyses with T1 and T2 STIR sequences are sufficient in routine
- Gadolinium injection is not recommended
- WB‐MRI is not recommended in routine

What are MRI limitations?

- Definition of “hypersignal” is subjective if T1/2 are not quantitatively measured (not in routine)
- Hypersignals may be an artifact and T2 STIR hypersignal is not specific for muscle inflammatory infiltrates
- One question one MRI protocol, WB MRI is not an all‐in‐one

## AUTHOR CONTRIBUTIONS

Samuel Malartre and Yves Allenbach contributed to the conception, writing and corrections. Damien Bachasson, Guillaume Mercy, Elissone Sarkis, Celine Anquetil and Oliver Benveniste contributed to the writing and corrections. All authors contributed to manuscript preparation and approved the final version.

## Data Availability

No original data were presented.
